# Where Did the Pericardial Effusion Go? A Case of Cardiopulmonary Resuscitation Acting as Treatment for Pericardial Tamponade

**DOI:** 10.1155/2021/9932485

**Published:** 2021-09-25

**Authors:** Varun tej Gonuguntla, Parita Soni, Nishil Dalsania, Ravi Karan Patti, Somal Navjot, Seneviratne Chanaka, Yizhak Kupfer

**Affiliations:** Maimonides Medical Center, 4802 10th Avenue, Brooklyn, NY 11219, USA

## Abstract

Pericardial tamponade results in multiple organ dysfunction and can lead to cardiac arrest. Cardiopulmonary resuscitation (CPR), a life-saving measure performed on patients in cardiac arrest, can lead to thoracic organ damage. However, CPR rarely acts as a therapeutic treatment for pericardial tamponade. Our case describes a patient admitted with pericardial tamponade in whom CPR provided therapeutic treatment with pericardial rupture and resolution of the tamponade.

## 1. Introduction

CPR is a life-saving measure typically involving repetitive external thoracic pressure causing direct compression to intrathoracic organs with the intent to provide circulatory support. Effective CPR is well known to have complications, mainly due to internal and external thoracic injuries. Common damage involves tracheal and bony cartilaginous chest structures, while injuries to the pleura, pericardium, myocardium, and intra-abdominal organs are typically rare [1]. This article portrays an interesting case of an elderly man who presented with a large pericardial effusion with tamponade, which completely resolved after life-saving therapeutic and effective CPR.

## 2. Case

A 70-year-old man with a medical history of diabetes mellitus, hypertension, hyperlipidemia, resolved COVID-19 infection, and gout presented to the emergency department (ED) with abdominal distention and shortness of breath. The patient's symptoms began as right upper quadrant pain and decreased appetite four days prior to presentation. He denied any temporal relationship with food, history of gallstones, or peptic ulcer disease. He also denied any fevers, chills, chest pain, nausea, vomiting, or changes in bowel movements. In the ED, his vitals were significant for oxyhemoglobin saturation of 87% on room air improving to 94% with 2 liters nasal cannula, blood pressure was 105/73 mmHg, heart rate was 88 beats per minute, and he was afebrile. His physical exam was significant for cold cyanosis of the fingers and toes and tenderness to palpation of the right upper quadrant abdominal area, and chest auscultation did not reveal any significant murmurs or muffled heart sounds.

**Figure 1:**
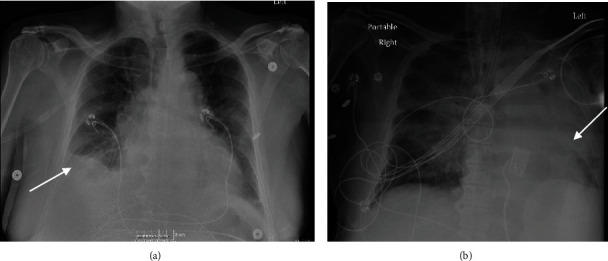
(a) Chest radiograph showing small right pleural effusion on admission. (b) Chest radiograph post-CPR showing large left pleural effusion.

**Figure 2:**
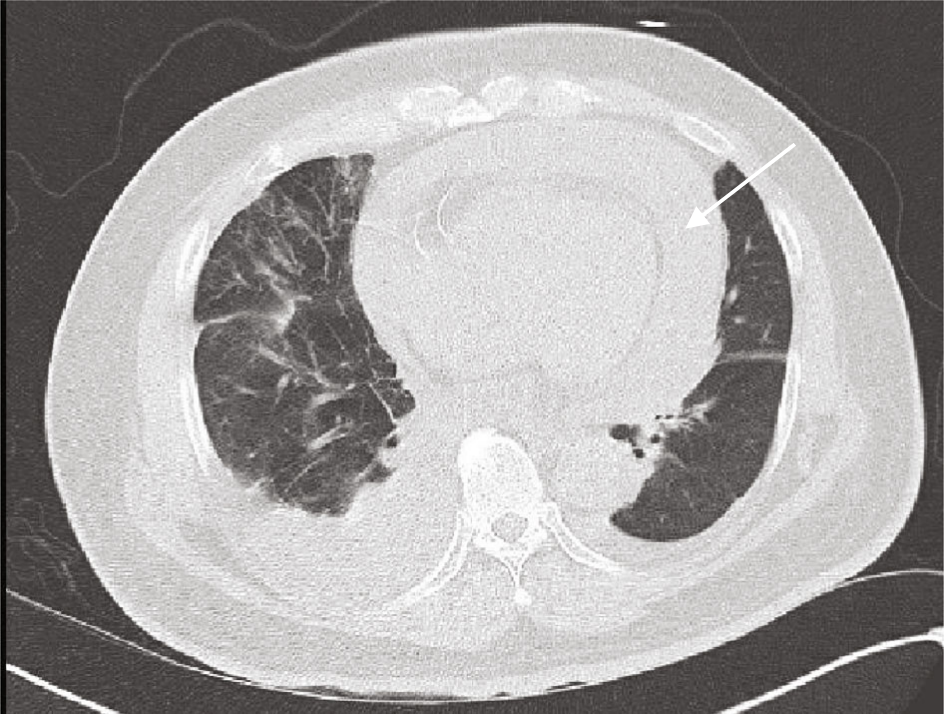
Computer tomography scan on admission with large pericardial effusion.

**Figure 3:**
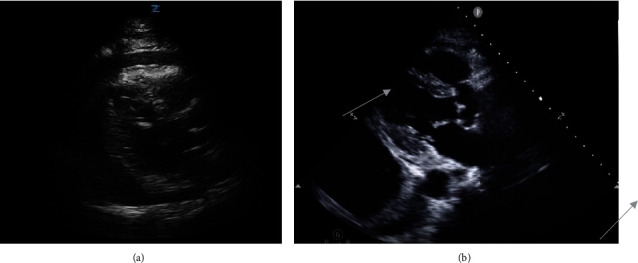
(a) Large pericardial effusion prior to cardiac arrest. (b) Post-CPR resolution of pericardial effusion and large left pleural effusion.

Initial labs were significant for severe metabolic acidosis, lactic acidosis, acute renal failure, hyperkalemia, and severe transaminitis. On imaging, chest radiograph (CXR) portrayed bilateral pulmonary vascular congestion with small bilateral pleural effusions (Figure 1(a)). Computerized tomography (CT) scan of the chest revealed large pericardial effusion and small bilateral pleural effusions right greater than the left side (Figure 2). CT scan of the abdomen and pelvis showed small volume ascites and mesenteric edema. Electrocardiogram portrayed diffuse low voltage. Bedside transthoracic echocardiogram confirmed moderate to large pericardial effusion with right ventricular collapse concerning for tamponade (Figure 3(a), Video 1).

The patient was admitted to the medical intensive care unit for management of multiorgan failure in the setting of pericardial effusion concerning for cardiac tamponade, and cardiothoracic surgery was consulted for evaluation for pericardiocentesis. He was adequately hydrated with sodium bicarbonate containing intravenous fluids, and without improvement of hemodynamics, a norepinephrine drip was started. After transfer to the intensive care unit, he was noted to have quickly progressive worsening respiratory distress, tachycardia, hypotension, dilated neck veins, and subsequently became pulseless. Immediate CPR was initiated with return of spontaneous circulation within 15 minutes. Postcardiac arrest labs showed worsening metabolic acidosis with increasing lactic acidosis. However, the patient's vital signs post-CPR improved significantly and he was able to be quickly titrated off norepinephrine.

The patient's CXR postresuscitation showed worsening left pleural effusion without pneumothorax (Figure 1(b)). Transthoracic echocardiogram postresuscitation revealed left ventricular ejection fraction of 61-65% with near complete resolution of the pericardial effusion and new left pleural effusion (Figure 3(b), Video 2). Left pleural percutaneous chest tube was placed with drainage of serosanguinous exudative fluid consistent with hemothorax per pleural fluid criteria. The patient's hospital course was complicated with progressively worsening multiorgan dysfunction despite medical management, and the patient's family opted for comfort measures and subsequently he died.

## 3. Discussion

Cardiac tamponade is the result of significant pericardial effusion between the visceral and parietal pleura of the heart causing compressive effects on the myocardium leading to diminished cardiac filling [1]. The low cardiac filling results in decreased cardiac output with resultant multiorgan dysfunction. Early detection of cardiac tamponade is important as it can lead to significant cardiac compromise and sometimes death. Diagnosis of cardiac tamponade is made from a high index of suspicion from clinical history and physical exam, which is then confirmed by echocardiogram showing right ventricular collapse from pericardial effusion [2]. In this case, our patient presented with progressive cardiac tamponade with subsequent volume overload, hepatic dysfunction, renal failure, hypotension, and further cardiac arrest.

Adequate chest compression and recoil is the hallmark of proper CPR, and it is well documented that CPR can result in damage to the thoracic as well as abdominal structures [3–5]. The structures that are damaged and the severity of damage depend on if it is manual compressions or active compression decompression (ACD) CPR devices. According to one study, the incidence of pericardial injury occurs in approximately 9% of the cases of CPR [3]. There is only handful of cases where the pericardium was injured due to CPR [6, 7]. However, the exact mechanism of this injury to the pericardium is poorly studied. The proposed mechanism of injury to the pericardium involves damage to the skeletal structures of the thorax. Rib fractures occur in 25% of the cases in manual compressions and as high as 93% if ACD-CPR device is involved [8]. The repeated compressions during CPR can result in the trauma to the viscera from the fragments of the bony ribs.

However, there are few cases where there is pericardial damage without rib fracture(s) [9, 10]. In both these cases, patients had hemopericardium 2/2 injury to ventricular wall and ruptured thoracic aortic aneurysm which were salvaged due to tear in pericardium after CPR. The proposed mechanism in these cases was increased pressure in the pericardium due to hemopericardium. The external compressive force exerted on the pericardium during CPR lead to the rise of pericardial tension, leading to pericardial rupture and consequently pericardial fluid draining into the pleural space.

In our patient, effective CPR likely led to pericardial rupture with draining of the pericardial effusion into the contiguous pleural space leading to the resolution of pericardial effusion [11, 12]. The post-CPR CXR did not show any fractured ribs but portrayed a large left pleural effusion not initially seen on initial CXR or on CT scan of chest. He also had resolution of pericardial effusion on transthoracic echocardiogram and subsequent improvement of hemodynamics. Therefore, in our case, effective CPR with pericardial rupture led to similar therapeutic benefits of pericardiocentesis or pericardial window. To our knowledge, very few cases have been reported in the medical literature where CPR has led to complete resolution of pericardial tamponade.

## 4. Conclusion

In conclusion, good-quality chest compressions are imperative for effective CPR, but rupture of the pericardium is a known complication. Although the possible complication of pericardial rupture with CPR does not suggest a change in the management of pericardial tamponade, a post-CPR evaluation of a patient with a previously known pericardial effusion or tamponade is important.

## Data Availability

Data will be available on request.

## Supplementary Materials

Supplementary Materials Video 1: echocardiogram of patient in the emergency room showing pericardial tamponade. Video 2: echocardiogram of patient post-CPR showing resolution of pericardial tamponade with new large pleural effusion. https://drive.google.com/drive/folders/1mt1Qct2WybVueQpb1AqoTozPPZkA_THl?usp=sharing
