# Supplementary material for: Where Did the Pericardial Effusion Go? A Case of Cardiopulmonary Resuscitation Acting as Treatment for Pericardial Tamponade
Source: Case Rep Crit Care. 2021 Sep 25;2021:9932485. doi: 10.1155/2021/9932485 (PMC8487396; doi:10.1155/2021/9932485)
Supplement: Supplementary Materials — Video 1: echocardiogram of patient in the emergency room showing pericardial tamponade. Video 2: echocardiogram of patient post-CPR showing resolution of pericardial tamponade with new large pleural effusion. https://drive.google.com/drive/folders/1mt1Qct2WybVueQpb1AqoTozPPZkA_THl?usp=sharing [file 9932485.f1.docx]

**Supplementary Material:**

https://drive.google.com/drive/folders/1mt1Qct2WybVueQpb1AqoTozPPZkA_THl?usp=sharing
